# Recent advances in incretin-based therapy for the treatment of cognitive impairment associated to the type 2 diabetes mellitus: preclinical and clinical studies - a narrative review

**DOI:** 10.3389/fendo.2025.1696419

**Published:** 2025-12-17

**Authors:** Federica Giofrè, Isabella Zaffina, Maria Chiara Pelle, Franco Arturi

**Affiliations:** 1 Unit of Internal Medicine, Department of Medicine, Hospital of Vibo Valentia, Vibo Valentia, Italy; 2 Internal Medicine Unit, San Francesco Hospital, Paola, Cosenza, Italy; 3 “Renato Dulbecco” University Hospital, Catanzaro, Italy; 4 Internal Medicine, Department of Science, Magna Graecia University, Catanzaro, Italy; 5 Research Centre for the Prevention and Treatment of Metabolic Diseases (CR METDIS), “Magna Graecia” University of Catanzaro, Catanzaro, Italy

**Keywords:** incretines, diabetes, dementia, cognitive impairment, diabetic complications

## Abstract

It is well-established that individuals with type 2 diabetes mellitus (T2DM) have an increased risk of developing cognitive impairment and dementia, suggesting a close relation between hyperglycemia, insulin resistance, and chronic inflammation. This decline is characterized by a large variety of symptoms going from mild to major form of cognitive impairment characterized of loss of memory, attention, processing speed, and executive function. Preserving the physiological level of glycemia improves cognitive performance, but untreated or inadequately diabetes therapy facilitates the risk of dementia. Some experimental studies have disclosed that drug for diabetes can have protective outcomes on cognitive impairment. In this context, incretin hormone glucagon-like peptide-1 (GLP-1) can reduce blood glucose, improve glucose transport through cell membranes, and to improve brain insulin resistance modulating neuroinflammation. In fact, GLP-1 acts as a neurotransmitter and neuromodulator activating central GLP-1 receptors located in the neurons determining its neurotropic and neuroprotective role in central nervous system. Preclinical and clinical studies suggest the potential role of dipeptidyl peptidase-4 inhibitors (DPP4-i) as therapy for the treatment and prevention of cognitive impairment and dementia. Similarly, several evidences demonstrated that treatment with glucagon-like peptide-1 receptor agonists (GLP-1 RAs) reduces the risk of cognitive impairment and dementia in T2DM patients by improving learning, memory, attention and executive functions. In addition, preclinical studies suggest a possible neuroprotective effect of GLP-1/GIP dual receptor agonist in animal models. The current narrative review, including studies published from September 1987 to September 2025, summarized the recent improvements regarding to the incretin-based therapy for cognitive impairment associated to the type 2 diabetes mellitus.

## 1 Introduction

**Figure 1:**
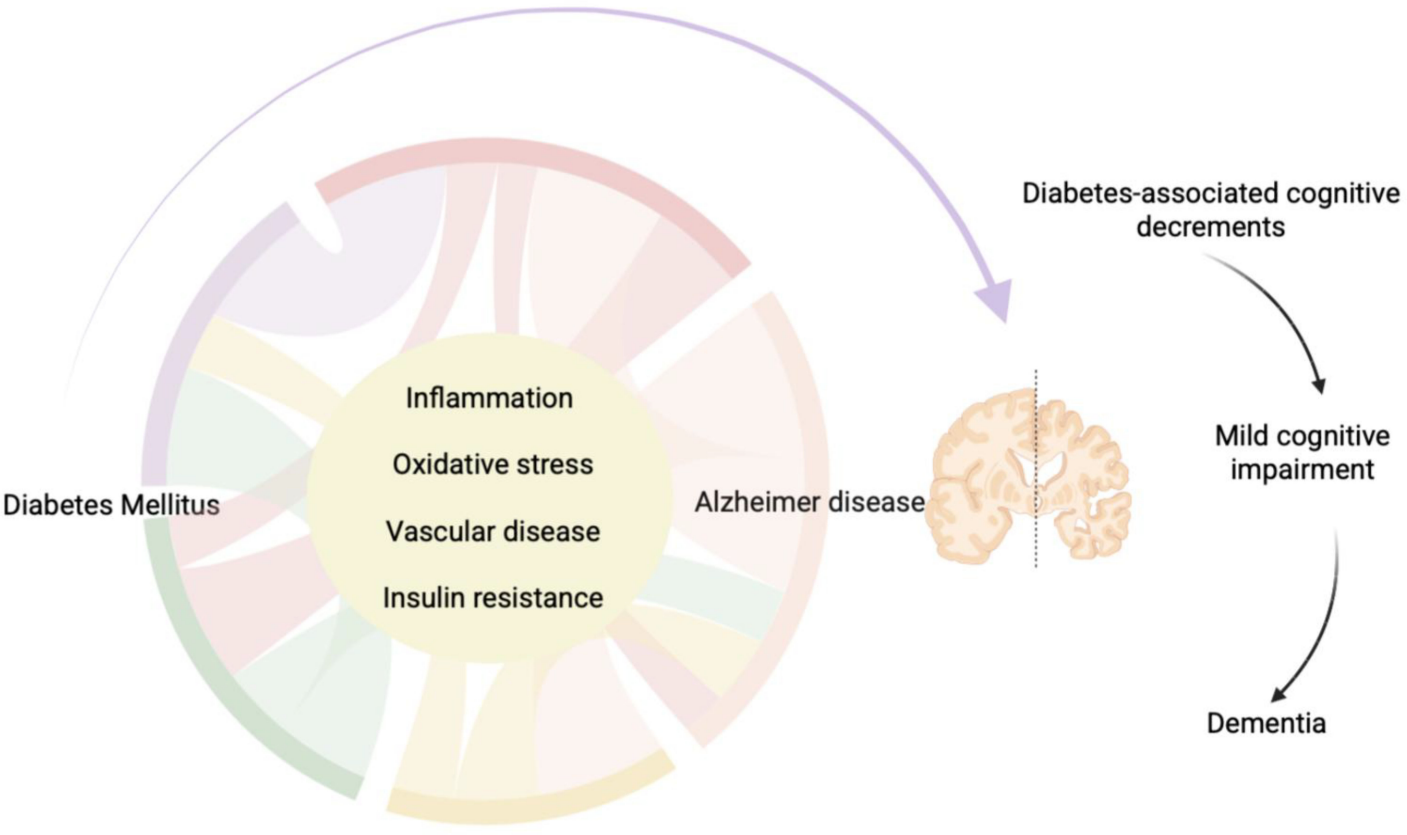
The relationship between diabetes mellitus and Alzheimer disease. The connection between diabetes and brain health has elicited interest within the scientific community demonstrating that T2DM pathogenetic mechanisms may overlap those of dementia, like inflammation, oxidative stress, vascular disease, and insulin resistance. Moreover, AD may represent a brain-specific diabetes mellitus called type 3 diabetes with three phases: diabetes-associated cognitive decrements, mild cognitive impairment (MCI), and dementia.

Type 2 diabetes mellitus (T2DM) represents one of the most significant challenges in modern medicine, with an increasing prevalence worldwide. According to International Diabetes Federation 589 million adults (20–79 years) are living with diabetes and this number is predicted to rise to 853 million by 2050 (1). This metabolic condition, characterized by insulin resistance and chronic hyperglycemia, not only leads to known cardiovascular, renal, and vascular complications but has also been associated with an increased risk of cognitive decline and dementia, such as Alzheimer’s disease (AD) (2). Numerous epidemiological and clinical studies have shown that patients with T2DM are more susceptible to deteriorating cognitive functions, with consequences that significantly impact quality of life and independence (3). Indeed T2DM pathogenetic mechanisms may overlap those of dementia, like inflammation, oxidative stress, vascular disease, and insulin resistance (4). Even some authors suggest that AD may represent a brain-specific diabetes mellitus called type 3 diabetes (5). It should be noted that there is a high risk of cerebrovascular disease in T2DM, and these vascular alterations can determine cognitive impairment and dementia (6). Also, it’s well known that insulin resistance supports the accumulation of β-amyloid (Aβ) and aberrant tau phosphorylation, main pathogenetic processes of AD (7). Cognitive decline in diabetes is not only a concomitant condition because it has also implications on daily management of T2DM, such as the adherence to medication, the ability of self-monitoring of glucose and of adjusting insulin doses to avoid hyper- and hypoglycaemia (8). The connection between diabetes and brain health has sparked intense interest within the scientific community (9), prompting research into innovative therapeutic strategies that can modify also the T2DM neurological complications. In this context, incretin-based therapies have emerged as one of the most promising frontiers with neuroprotective, anti-inflammatory, and neurorestorative effects, opening new perspectives for treating cognitive decline in patients with T2DM (10). It must also be noted that both GLP-1RA and DPP4-i have introduced a more physiologic approach to glycemic regulation minimizing the risk of hypoglycemia—a complication still common with other drugs. This safety profile has important neuroprotective implications, because recurrent hypoglycemic episodes are an independent risk factor for cognitive decline and dementia in older adults (11, 12). Incretin therapies may indirectly preserve neuronal integrity and reduce metabolic stress on the central nervous system by promoting a stable glycemic profile. Obviously, the last clinical decision should come from a balance between these benefits and potential side effects, such as gastrointestinal intolerance, unintended weight loss in frail individuals, pancreatitis and potential changes in the exocrine pancreas, loss of appetite, headache, dizziness, and drug–drug interactions (13) and considering drawback in their use: advanced chronic kidney disease, history of acute pancreatitis, severe gastrointestinal disorders (14). It’s necessary to underline that in diabetic cognitive dysfunction we find three phases: diabetes-associated cognitive decrements, mild cognitive impairment (MCI), and dementia (**Figure 1**) (15).

The present review summarizes improvements regarding the incretin-based therapy for cognitive impairment associated to the type 2 diabetes mellitus, discussing studies published in literature in a period from September 1987 through September 2025.The goal is to provide an updated overview of the potential of these innovative treatments, helping to outline a future path that could improve the integrated management of T2DM and its neurological complications.

## 2 Diabetes and cognitive impairment

There are several scientific evidences that diabetes and prediabetes predispose to cognitive decline leading to dementia, with reduced performance on multiple domains of cognitive function (16). Cognitive impairment (CI), starting from MCI leading to dementia, is characterized by memory impairment, reduced psychomotor function, affected verbal fluency and attention and is yet associated with functional and structural changes in the brain (17). Dementia is a chronic, irreversible condition characterized by preserved consciousness, but mental instability, memory loss, disrupted emotional control (18). In 20 to 30% of cases, vascular dementia occurs in individuals with diabetes. Importantly, there are differences between type 1 diabetes mellitus (T1DM) and T2DM: the first one appears to be associated to a slow-down of mental processing and executive/attentional functioning, above all in patients with an early onset of diabetes, but remains stable over the years, while the second one has additionally memory problems. Moreover, T1DM is diagnosed during childhood and adolescence, when brain is in period of development, so is more susceptible to hyperglycemia. In patients with T1DM, glycemic control appears to play a role in cognitive performance. In fact, the Diabetes Control and Complications Trial (DCCT) revealed a better result on tests of motor speed and psychomotor efficiency in patients with T1DM with a time weighted mean glycated haemoglobin (HbA1c) less than 7.4%, than those subjects whose time weighted mean HbA1c was greater than 8.8%. Moreover, the age of onset of T1DM may also contribute to the presence of cognitive impairment, early diagnosis at less than 4 years of age is associated with a poor executive skills, attention, and processing speed, compared with those that were diagnosed after 4 yr of age (19). In most cases, T2DM is diagnosed in adulthood, and older age itself is a risk factor for cognitive decline, in fact the cognitive alterations observed in T2DM is similar to the alteration observed in cognitive aging and has a negative impact on daily life (20). In T2DM the first stage of cognitive dysfunction may not affect daily life and involves brain speed and memory. The second stage is MCI (non-amnesic MCI and amnesic MCI), includes declines in some cognitive domains with a subtle impact on daily life; diabetes increases the risk to develop amnesic and non-amnesic MCI. Epidemiological studies showed hazard ratios (HRs) of 1.5 and 1.2 for amnesic MCI and non-amnesic MCI, respectively in T2DM population. The last stage is dementia and involves many cognitive domains with severe consequences in daily life and self-care. Moreover, T2DM increases incidence of Alzheimer’s disease and vascular dementia (VaD) (21). Both in T1DM and T2DM it was demonstrated an association between the cognitive impairment and diabetes complications such as proliferative retinopathy, hypertension and polyneuropathy. In patients with T2DM brain imaging reveals alterations, above all there is a loss of white matter in the frontal and temporal regions and of gray matter in the medial temporal, medial frontal lobes and anterior cingulate area.

### 2.1 The pathophysiology of cognitive dysfunction in diabetes

The pathophysiology of cognitive dysfunction in diabetes is not completely clear. Many evidence supported a potential causative role for vascular disease, hyperglycemia, hypoglycemia, insulin resistance and amyloid deposition. Individuals with diabetes have a 2–6 times higher risk of thrombotic stroke (22), and vascular disease appears to contribute to abnormalities in cognition in such patients. In patients with diabetes, an overall decrease in cerebral blood flow has also been demonstrated, the degree of severity is related to the duration of the disease (23). As in other organs, also in the brain hyperglycemia can induce alterations by increasing polyol pathway activation, formation of advanced glycation end products (AGEs), diacylglycerol activation of protein kinase C, and shifting glucose into the hexosamine pathway (24, 25). Animal studies showed an increased expression of AGEs and receptors for AGE (RAGEs) in glial cells and neurons and damage to white matter and myelin in diabetic mice (26). In post-mortem human studies, individuals with diabetes and Alzheimer’s disease showed to have greater N-carboxymethyllysine (a type of AGE) staining on brain slices (27), and had a difference in the quantity of AGE-like glycated protein rich neurofibrillary tangles and senile plaques (28). Aragnoet al. (29) showed that galectin-3 (a proatherogenic molecule), RAGEs and the polyol pathway activation were increased in rat brains, while S-100 protein, a marker for brain injury that can bind to RAGEs, and Nuclear factor κB (NF-κB) transcription factors, a proinflammatory gene marker up-regulated by AGEs, were both up-regulated in the hippocampus. Moreover, in diabetic rats neurochemical changes have been observed, including an impairment of long-term potentiation (30), decreased dopamine and acetylcholine activity (31), decreased serotonin turnover and increased norepinephrine (32), suggesting a worsen neurotransmitter function. It is known that also repetitive episodes of moderate to severe hypoglycemia play a possible role in etiology of cognitive dysfunction in the young (33). In human patients who died of hypoglycemia, laminar necrosis and gliosis found in cortex, basal ganglia, and hippocampus (34). In other post-mortem human studies, multifocal or diffuse necrosis of the cerebral cortex has been demonstrated (35). Also insulin resistance contribute in impairment of cognitive function. In fact insulin plays an important role in brain, it is involved in regulation of glucose metabolism, growth of neurons, cognitive function. Alterations of insulin signalling may accelerate brain aging, impair plasticity, induce possibly neurodegeneration, alter permeability to insulin of the blood–brain barrier (BBB) (36), and downregulate the level of insulin receptors on the endothelium of the cerebral blood vessels (37). Moreover, it is now known that insulin has a neuroprotective action, preventing the formation of toxic beta-amyloid (Aβ), a component of amyloid plaques, a hallmark of AD (38). Several data suggested that insulin resistance leads to an Aβ accumulation, through increasing the activity of β-site amyloid precursor protein cleaving enzyme -1 (BACE-1), which is responsible for the proteolysis of amyloid precursor protein (APP) (39–41), reducing the activity of insulin-degrading enzyme (IDE), which is involved in the degradation of β-amyloid and the intracellular domain of APP. B-amyloid accumulation maintains insulin resistance, through serine phosphorylation of insulin receptor substrate- 1 (IRS-1) (5). Moreover, solubilized amyloid-β can activate pro-inflammatory pathways, that amplify vasoconstriction and vascular inflammation (42). Insulin resistance promotes the hyperphosphorylation of the tau protein, which aggregated in intracellular neurofibrillary tangles (NFTs), resulting in progressive neuronal loss and neurodegeneration, especially in the hippocampus and olfactory epithelium (43). Several studies demonstrated that brain insulin resistance occur also in patients with AD and without T2DM, suggesting that insulin resistance in brain depends on glucose independent mechanisms (44). In the post-mortem brains from AD patients, De la Monte et al. (45, 46) showed decreased expression of insulin receptors, insulin-like growth factors 1 and 2 (IGF1 and IGF2), Insulin receptor substrate 1(IRS1), phosphatidylinositol 3-kinase/Akt (PI3K/Akt) and increased Glycogen Synthase Kinase 3 beta (GSK-3β) level. These alterations changes were inversely correlated with severity of AD, which indicates the relationship between impaired insulin signalling and progression of the disease (47).

## 3 Role of GLP-1 and GIP in central nervous system

Incretin-based therapies represent a novel treatment for T2DM, relying on the insulinotropic actions of the gut hormone GLP-1 and, most recently, on the combined action of GLP-1 and the gastric inhibitory polypeptide (GIP) hormones. Incretins are gastrointestinal hormones that promote postprandial insulin secretion in a glucose-dependent manner. There are two types of incretins: GLP-1 and GIP. Both are secreted by intestinal cells, with GLP-1 being secreted from L cells that are found in the large and lower bowel (48), and GIP from K cells in the upper bowel. GLP-1 subsequently binds to GLP-1 receptors (GLP-1R), which are widely expressed in multiple organs and tissues, including the gastrointestinal tract, the endocrine pancreas, the heart, and the central nervous system, thereby exerting pleiotropic functions. Incretin drugs have pleiotropic effects that enrich neurogenesis, decrease apoptosis, protect neurons from oxidative stress, and reduce neuroinflammation in various neurological conditions.

### 3.1 GLP-1

**Figure 2:**
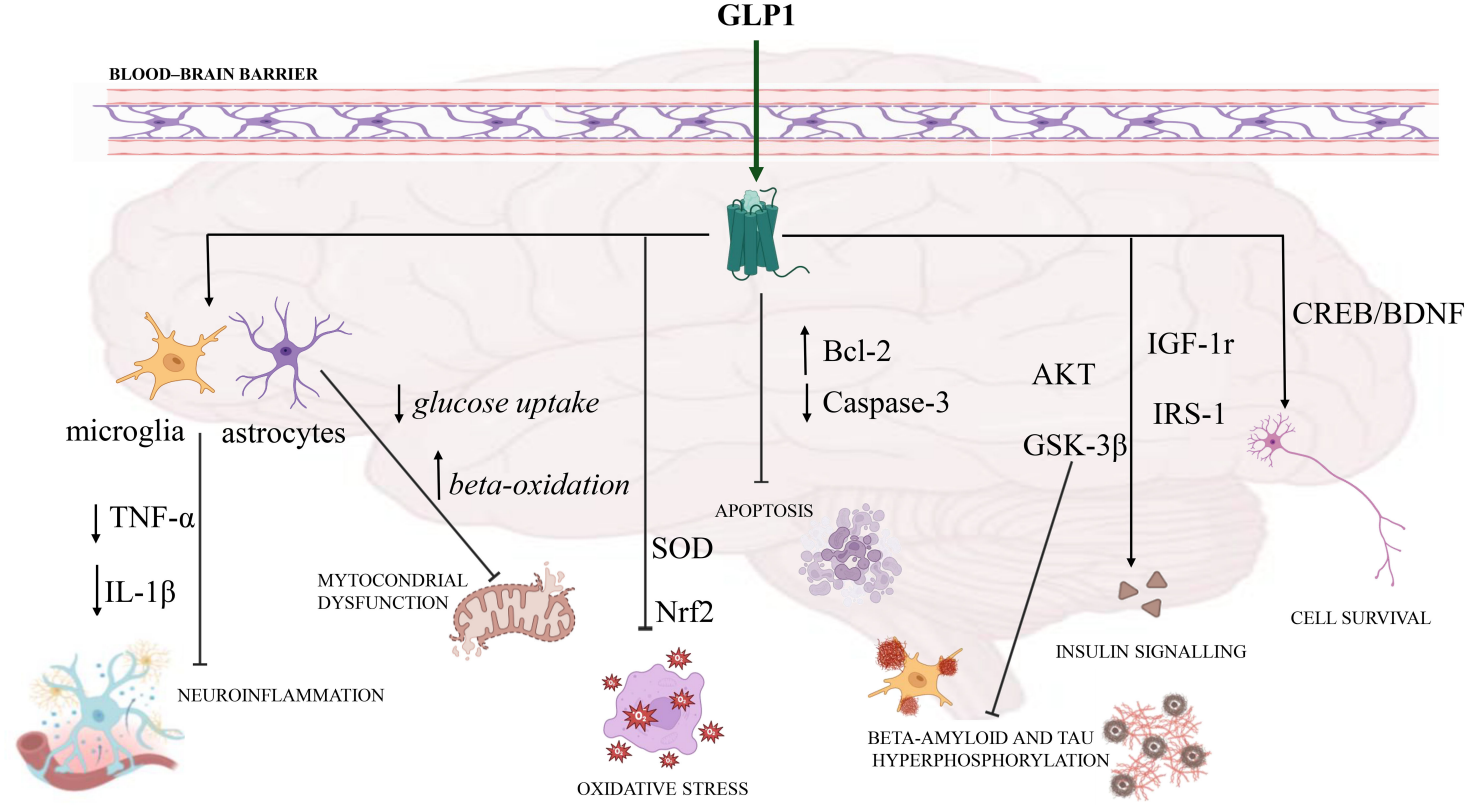
Role of GLP-1 in central nervous system. Activation of GLP-1R in the brain can modify numerous cellular processes such as neuroinflammation, oxidative stress, apoptosis, mitochondrial dysfunction, cell survival, insulin signalling, and neuronal functions. GLP-1 may mitigate the inflammatory response in the brain by reducing levels of pro-inflammatory cytokines such as tumor necrosis factor-a (TNF-a) and interleukin-1β (IL-1β) released by microglia and astrocytes. In addition, the loss of GLP-1R iN astrocytes can impair mitochondrial function. GLP1ra reduces oxidative stress through stimulation of antioxidant enzymes such as superoxide dismutase (SOD) and catalase via the erythroid nuclear factor 2 (Nrf2) pathway. It addition, it may inhibit apoptosis by increasing anti-apoptotic B-cell lymphoma 2 (Bcl-2) and reducing caspase-3 activation. Additional actions are neurogenesis and synaptic plasticity through the cAMP response element (CREB)/brain derived neurotrophic factor (BDNF) signalling pathway. In addition, the neuroprotective effects of GLP-1RA may be a consequence of increased insulin signalling in brain cells, leading to improved insulin sensitivity in neurons through the insulin receptor-1 substrate (IRS-1), AKT, and GSK-3B pathways. Finally, GSK-3β participates in neuroprotection by reducing beta-amyloid levels and tau hyperphosphorylation in AD pathology.

Several evidences indicate that GLP-1receptor agonist can cross the blood–brain barrier and GLP-1Rs are localized is stated in the central nervous systems, including the frontal and occipital lobes, cerebellum, substantia nigra, the hippocampus, thalamus and hypothalamus (49–51). Activation of GLP-1R in the brain can regulate numerous cellular processes such as neuroinflammation, oxidative stress, apoptosis, and mitochondrial dysfunction, and may promote cell survival, restore insulin signalling and improve neuronal functions (52–54). Neuroinflammation is a central pathophysiological factor of neurodegenerative disorders and GLP-1 can mitigate inflammation response in the brain by reducing levels of pro-inflammatory cytokines such as Tumor necrosis factor-α (TNF-α) and Interleukin-1β (IL-1β) liberated by microglia and astrocytes (55, 56). The last cells are the most abundant type in adult brain tissue, and it has newly been documented for their crucial part in controlling glucose and energy homeostasis (57–60). Therefore, the loss of GLP-1R in astrocytes can harm mitochondrial function, because GLP-1 prevents glucose uptake in astrocytes and stimulates beta-oxidation, that is important for energy balance in the brain and mitochondrial integrity (59). Furthermore, studies suggest that GLP1ra diminished oxidative stress via stimulation of antioxidant enzymes such as superoxide dismutase (SOD) and catalase through nuclear factor erythroid 2-related factor 2 (Nrf2) pathway (60). Moreover, being GLP-1 a growth factor, it stimulates growth of neurites, repair and replacement of cells, increases cell metabolism, and reduces apoptosis (61). In fact, inhibition of apoptosis is mediated by increase of anti-apoptotic B-cell lymphoma 2 (Bcl-2) and reduction of caspase-3 activation (62). Additionally, GLP1ra stimulates mitochondrial biogenesis and function via modulation of peroxisome proliferator-activated receptor gamma coactivator 1-alpha (PGC-1α) and sirtuin 1 (SIRT1) pathway, enhancing energy metabolism and decreasing reactive oxygen species (ROS) production (63). These drugs can induce neurogenesis and synaptic plasticity through cAMP-response element binding protein (CREB)/brain derived neurotrophic factor (BDNF) signalling pathway, thus promoting neuronal survival and synaptic functions (64, 65). Moreover, the neuroprotective effects of GLP-1RAs can be a consequence of the increased insulin signalling in the brain cells, leading to improved insulin sensitivity in the neurons (52). In the brain, GLP-1 can induce insulin release, through over-expression of the internalized insulin and IGF-1 receptors (55). Exendin-4 (Ex-4), a GLP-1RA, improved insulin resistance in neurons through insulin receptor substrate-1 (IRS-1), AKT, and GSK-3β pathways. GSK-3β participates to neuroprotection by reducing the levels of beta-amyloid and tau hyperphosphorylation in AD pathology (66). Tau protein is crucial for microtubule functions and glucose metabolism, but when it becomes hyperphosphorylated by specific kinases such as GSK-3β and cyclin-dependent kinase 5 (CDK5), loses its capacity to bind to microtubules, with consequent aggregation within neurons and in the extracellular space (**Figure 2**) (67–70). And above all, the Ex-4 modulates the tau hyperphosphorylation and cognitive dysfunction by raising the *Ins2*-derived brain insulin via Wnt/β-catenin/NeuroD1 pathway in T2DM (71). Lastly, GLP-1RAs can improve memory and learning (72).

### 3.2 GIP

The biological action of GIP is promoted through binding to the GIP receptor (GIPR), a class B G-protein-coupled receptor that is similar to GLP-1R and belongs to the glucagon receptor family. The GIPR is expressed in the endocrine pancreas, in adipocytes, in myeloid cells, in the endothelium of the heart and blood vessels, the inner layers of the adrenal cortex and in the central nervous system (48).

**Figure 3:**
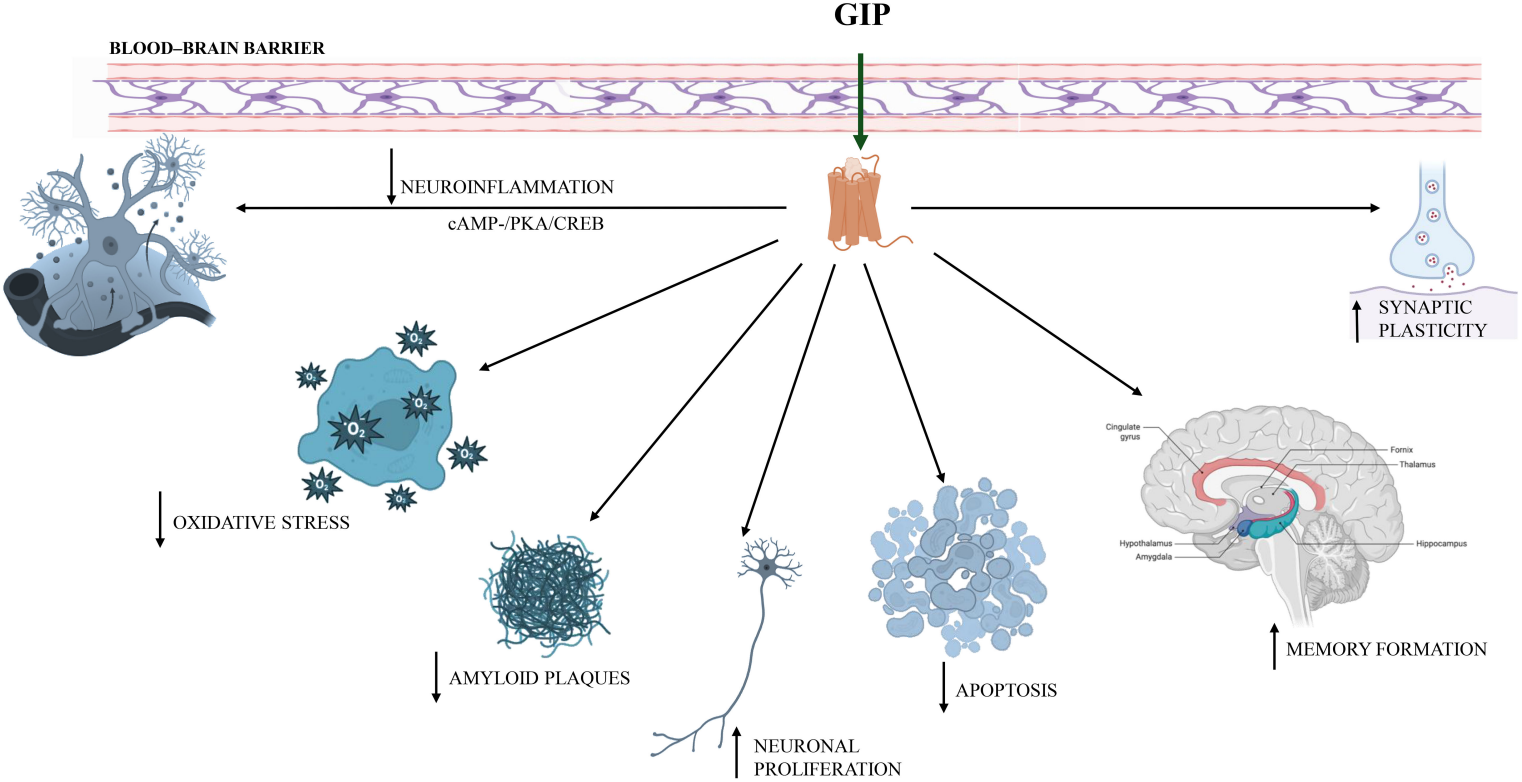
Role of GIP in central nervous system. GIP can cross the blood brain barrier and can stimulate neuronal, decreasing both amyloid plaques in the cortex and the neuroinflammation by upregulating cAMP-/PKA/CREB signalling pathway. Additionally, GIP can act on memory formation and synaptic plasticity as well as inhibition of apoptosis of cerebellar cells. The effect of GIP on redox balance is mediated by a decreasing of reactive oxygen species and releasing of nitric oxide.

Indeed, similar to GLP-1R, GIP receptors have been detected in many areas of the brain including the hippocampus and hippocampal progenitor cells. GIP and its agonists can cross the blood brain barrier and present significant neuroprotective effects on synapse function and numbers. They can stimulate neuronal proliferation, decreasing both amyloid plaques in the cortex and the chronic inflammation (73). Additionally, GIP analogues can act on memory formation (74–76) and synaptic plasticity and, being GIP a neurotrophic factor, it can inhibit apoptosis of cerebellar cells (77). A novel long-acting GIP analogue exhibited to alleviate central pathological development in AD mice, with the underlying mechanism concerning to the inhibition of neuroinflammation and the upregulation of cAMP-/PKA/CREB signalling pathway (78). Systemic anti-inflammatory effects of GIP are related to reduced mRNA expression of macrophage chemoattractant protein 1 (MCP-1), vascular cell adhesion molecule 1 (VCAM-1), and intercellular adhesion molecule 1 (ICAM) together with decreased circulating levels of interleukin IL-1β and TNFα (79). The effect of GIP on redox balance is showed as decreased reactive oxygen species and release of nitric oxide (**Figure 3**) (80).

### 3.3 Dipeptidylpeptidase IV (DPP-4)

However, GLP-1 and GIP are rapidly degraded in plasma by dipeptidyl peptidase IV (DPP-4), resulting in a very short half-life of approximately 1.5 minutes (81). DPP-4 is a type-II integral transmembrane glycoprotein with enzymatic activity; it also exists as a soluble form in the plasma, lacking the cytoplasmic and transmembrane domains. The primary substrates for DPP-4’s catalytic activity are incretins responsible for glucose metabolism, including GIP, GLP-1 and GLP-2 (82, 83). Consequently, DPP-4 inhibitors (DPP4-i) may have a pivotal role in prolonging the circulating half-life of GLP-1 and GIP, thereby preventing their cleavage (84).

## 4 Role of DPP-4 inhibitors in cognitive disfunction in T2DM

Incretin-based therapies represent a novel treatment for T2DM, relying on the insulinotropic actions of the gut hormone GLP-1 and, most recently, on the combined action of GLP-1 and the GIP hormones. Furthermore, the dipeptidyl peptidase-4 inhibitors (DPP4-i) are a class of anti- diabetic drugs that enhances the blood concentration of active GLP-1 (85) also are able to improve glucose metabolism. However, in recent years, researchers have begun to explore their potential neuroprotective effects and the role they might play in slowing down or preventing cognitive decline, especially in the context of neurodegenerative diseases like Alzheimer’s. In fact, DPP4 is a cell surface glycoprotein expressed in different cells such as vascular epithelial cells, immune cells and neurological cells (microglia, neurons, and astrocytes), so its inhibition by DPP4-i may help to protect against dementia (86).

Preclinical and clinical studies suggest the potential role of DPP4-i, of GLP-1 receptor agonist and, most recently, of GLP-1/GIP dual receptor agonists as therapy for the treatment and prevention of cognitive impairment and dementia.

### 4.1 Preclinical studies

At the preclinical level, in animal models and laboratory settings, there is some promising evidence and many interesting insights. Several studies have shown that DPP4-i can cross the blood-brain barrier, allowing them to act directly within the brain, and influence neuroinflammatory processes, the aggregation of pathological proteins, and neurodegeneration (87). It is well known in literature that DPP4-i have neuroprotective effects and modulate pathological pathway.

Kosaraju et al. demonstrate that the DPP4-i saxagliptin reduces beta-amyloid, tau accumulation (main culprits of neurodegeneration) and inflammatory markers and increases hippocampal GLP-1 and memory retention in mouse models of Alzheimer’s disease. This occurs through modulation of clearance and production processes of these proteins. This effect is due to the improvement of GLP-1 levels and inverts behavioural deficits observed in AD (88).

On the side of inflammation, Zhuge et al. (2024) investigated the effects of the DPP-4 inhibitor linagliptin on mild cognitive impairment in mice, focusing on the regulation of microglia. They treated mice with either linagliptin or metformin for a defined period and analysed brain tissue for DPP-4 and markers of neuroinflammation levels and microglial polarization states. First of all, they proved that DPP-4 activity was enhanced in the hippocampus of middle-aged mice, primarily in microglia, but treatment with Linagliptin reduced the activation of M1 Macrofage-type (pro-inflammatory) microglia and promoted M2 Macrofage-type (anti-inflammatory) polarization. These protective effects appear to be partially mediated by macrophage inflammatory protein-1α (MIP-1α, a DPP-4 substrate) because conversely if MIP-1α was knocked out they were lost (87).

Interestingly, linagliptin does not easily cross the blood-brain barrier (BBB), but its neuroprotective effects may be partly due to increased GLP-1 activity, which can cross the BBB effectively (89). Studies in animal models of AD have shown that treatment with DPP-4 inhibitors (such as saxagliptin, vildagliptin, linagliptin, and sitagliptin) increases GLP-1 expression in the hippocampus and other various brain regions (88–91). In these areas, GLP-1 bind to its receptor (GLP-1R), reducing amyloid deposition, tau levels, microglial activation, and memory decline (92). The preclinical studies performed in type 2 diabetes mellitus using the DPP-4 inhibitors are summarized in the **Table 1**.

**Table 1:** Preclinical and clinical studies on cognitive function in T2D using DPP-4 inhibitors.

Study	Type of study	Intervention	Outcome	Results
Kosaraju, J. et al., (2013) (88)	Preclinical	Saxagliptin administered to rats with streptozotocin-induced Alzheimer's disease	Cognitive deficits, oxidative stress, neuroinflammation	Saxagliptin improved memory and learning, and reduced oxidative stress and brain inflammation.
Zhuge, F. et al., (2024) (87)	Preclinical	Linagliptin administered to aged mice	Cognitive function and microglial polarization	Linagliptin improved age-related cognitive impairment and favorably regulated microglial polarization toward the anti-inflammatory M2 phenotype.
Kosaraju, J. et al., (2017) (89)	Preclinical	Linagliptin administered to 3xTg-AD mice (Alzheimer's disease mouse model)	Cognitive deficits and Alzheimer's pathology (plaques and tangles)	Linagliptin improved cognitive function and reduced beta-amyloid deposition and phosphorylated tau.
Kosaraju, J. et al., (2013) (90)	Preclinical	Vildagliptin administered to streptozotocin-induced AD rats	Cognitive impairment and AD pathology	Improved cognitive performance and reduced Alzheimer’s-related pathological change
Kosaraju, J. et al., (2014) (91)	Preclinical	DPP-4 inhibition by Pterocarpus marsupium and Eugenia jambolana in streptozotocin-induced AD rats	Cognitive function, oxidative stress	Improved memory performance and antioxidant defense; reduced oxidative stress and cognitive deficits
Angelopoulou, E. et al., (2018) (92)	Preclinical	Review of DPP-4 inhibitors as potential Alzheimer’s therapeutics	Alzheimer’s pathology and therapeutic prospects	DPP-4 inhibitors mitigated neurodegeneration and cognitive decline in AD
Rizzo, M.R. et al., (2014) (93)	Clinical	DPP-4 inhibitors in aged diabetic patients with mild cognitive impairment	Cognitive impairment	Protective effect on cognitive decline; slowed progression of cognitive impairment
Jeong, S.H. et al., (2021) (85)	Clinical	DPP-4 inhibitor use in diabetic patients with AD-related cognitive impairment	Amyloid burden and cognitive impairment	Associated with reduced amyloid burden; potential cognitive benefit in diabetic patients with AD
Isik, A.T. et al., (2017) (94)	Clinical	Sitagliptinin elderly diabetic patients with/without Alzheimer's disease	Cognitive function	Improved cognitive functions observed in elderly diabetic patients regardless of AD status
Kumar R. et al., (2013) (95)	Clinical	Measurement of serum Sirtuin1 levels	Early detection biomarker for Alzheimer's disease	Sirtuin1 identified as a promising serum biomarker for early detection of Alzheimer's disease
Kornelius, E. et al., (2015) (96)	Clinical	Linagliptin in neuronal cells	Abeta-induced cytotoxicity and AMPK activation	Linagliptin attenuated Abeta-induced cytotoxicity via activation of AMPK, suggesting neuroprotection
Herskovits, A.Z., Guarente, L. (2014) (97)	Clinical	Review of SIRT1 role in neurodevelopment and brain aging	Neurodevelopment and brain senescence	SIRT1 plays a key role in brain development and aging processes, impacting neuronal health
Chong, Z.Z. et al., (2012) (98)	Clinical	Review of SIRT1 in oxidative stress disorders	Oxidative stress and related disorders	SIRT1 is a promising therapeutic target for diseases related to oxidative stress
Kim, YG. et al., (2018) (99)	Clinical	Use of DPP-4 inhibitors versus sulfonylureas	Risk of dementia in older diabetic patients	DPP-4 inhibitor use associated with a lower risk of dementia compared to sulfonylureas in older patients with T2D
Wu, CY. et al., (2020) (100)	Clinical	Use of metformin or DPP4 inhibitors in T2D patients	Memory decline in T2D patients with or without Alzheimer's	DPP4 inhibitors and metformin use were linked to differences in memory decline, influenced by APOE genotype status

### 4.2 Clinical studies

Besides preclinical studies, in literature we found many clinical evidence regarding protective neuronal effect of DPP4-i. Firstly a retrospective study demonstrated that two years of DPP-4 inhibitor use was associated with reduced cognitive decline in diabetic patients with mild cognitive impairment (MCI). Although the study didn’t include patients with Alzheimer’s, it provided important evidence for a potential neuroprotective effect of DPP-i (93). Another retrospective study in 2021 highlighted that DPP-4i use reduces amyloid deposition and improves long-term cognitive outcome in diabetic patients with diabetic Alzheimer disease–related cognitive impairment (85). Similarly a prospective observational study evidenced that Diabetic patients with Alzheimer’s treated with sitagliptin had significantly higher MMSE scores compared to those treated with metformin. This suggests a possible cognitive benefit of sitagliptin in this population (94). Furthermore, a study searched to explain the possible biological mechanism behind DPP-4 inhibitor linagliptin effect on AD. Serum Sirt1 levels are found to be significantly reduced in Alzheimer’s patients, and a potential role for Sirt1 as a biomarker in this condition has been proposed (95). Linagliptin treatment increased Sirt1 expression in the peripheral blood leukocytes of diabetic patients with Alzheimer’s disease (96). Sirt1 is an enzyme with known neuroprotective properties: enhances synaptic plasticity and memory (97), reduces Aβaccumulation, suppresses oxidative stress and neuronal loss (98). The role of DPP4-i has been compared also with sulfonylureas in a Korean diabetic patients’ cohort. Data analysis demonstrated that DPP4-i administration was associated with a 34% lower risk of all-cause dementia compared with sulfonylureas in older patients with type 2 diabetes. In particular DPP4-i use reduced risk of Alzheimer’s disease, but not vascular dementia, compared with sulfonylureas (99). Wu CY et al., in their study, searched to define which type of patients could profit from the use of DPP4-i. They evaluated how the use of metformin or DPP−4 inhibitors influences memory decline in patients with type 2 diabetes, comparing groups with: normal cognitive function (NC) and AD. Moreover, the role of the Apolipoprotein ϵ4 (APOE ϵ4) genotype was considered as a potential modifying factor. Indeed, Metformin administration was associated with better memory performance in normal cognitive function population, while in AD dementia population, DPP4 inhibitor use was associated with a slowdown of memory decline. Furthermore, APOE ϵ4 carrier status may predict better results of DPP4 inhibitors in normal cognitive individuals, and lower benefit of metformin in people with AD. So, these results also confirmed the need of a personalized antidiabetic therapy (100). The clinical studies performed in type 2 diabetes mellitus using the DPP-4 inhibitors are summarized in the **Table 1**.

## 5 Potential role of GLP-1RAs in cognitive dysfunction

Similarly, to the DPP4-1, also GLP-1 RAs have been widely studied (in preclinical and clinical context) for their neuroprotective mechanisms. Nowadays the approved GLP-1 RAs are exenatide, lixisenatide, liraglutide, dulaglutide and semaglutide (101). Actually besides the hypoglycemic effect, GLP-1 RAs seem to have neuroprotective effect improving cognitive dysfunction in individuals with or without diabetes (102). More research is needed to fully understand the real impact of GLP-1 RAs on cognitive decline, but their effects are promising in brain health reducing the risk of cognitive impairment.

### 5.1 Preclinical studies

Interest in GLP-1 agonists neurological effect begins in 2010 when McClean P.L. et al., demonstrated that liraglutide influences brain neurotransmission in rats and directly stimulates synaptic transmission in the hippocampus, enhancing long-term potentiation (LTP) (103). Similarly, Han et al. demonstrated that pre-treatment with liraglutide protects cognitive function (learning and memory) and reduces deficit of L-LTP induced by the bilateral intrahippocampal injection of amyloid- protein (25-35) in rats that had been administered amyloid-β (104). Also McClean P.L. et al. had previously demonstrated that periferical injection of liraglutide in Alzheimer mouse model (APP/PS1 mice) prevented memory deficits, reduced total amyloid plaques and dense-core plaques by 40–50%, and soluble oligomers by 25%, prevented synapse loss in the hippocampus and reduced the inflammation measured by microglial activation (reduced by 50%). These results suggested that use of liraglutide may be a promising strategy to prevent and ameliorate the cognitive function observed in AD. The effects of liraglutide was evaluated also on later stages of the AD (105). McClean P.L. et al. highlighted that liraglutide can reverse cognitive impairment, restore synaptic function, and reduce amyloid plaque burden even in later stages of the disease in aged APP/PS1 mice (mouse model of Alzheimer disease) (106). Moreover, it has been demonstrated that chronic liraglutide treatment stimulates neurogenesis in the hippocampus of Alzheimer’s model mice, suggesting it may help restore neuronal populations lost due to neurodegeneration (107). Long-Smith et al. showed that intraperitoneal injection of liraglutide for 8 weeks in mice improves impaired brain insulin signalling reduces amyloid plaques, neuroinflammation and insulin resistance in mouse model of AD (108). More recently, Palleria et al. treated rats, with diabetes induced by streptozotocin (STZ), with liraglutide for 6 weeks evaluating cognitive function with behavioural tests, metabolic parameters, brain oxidative stress, neuroinflammation, and neurotrophic factors (109). Liraglutide significantly improved learning and memory in diabetic rats, as shown by better performance in behavioural tests. These cognitive benefits occurred without major changes in blood glucose or body weight, indicating the effect was central (brain-related) rather than due to improved metabolic control. In addition, liraglutide reduced oxidative stress and inflammation in the brain and increased levels of BDNF (brain-derived neurotrophic factor), which supports neuronal health. Interestingly, several studies evaluated the effects of treatment with liraglutide on neuroinflammation, stress oxidative. Liraglutide uses another molecular pathway to ameliorate brain insulin resistance, in particular inhibiting the c-Jun N-terminal kinase (JNK) pathway and activation of Bcl2 gene in T2DM mice (110). A study, using *in vitro* and *in vivo* AD models, evaluated the effect of liraglutide in SH-SY5Y human neuroblastoma cell line by investigating tau activation and Beta-site amyloid precursor protein cleaving enzyme 1 (BACE-1) expression (BACE-1 is a transmembrane aspartate protease, namely a key enzyme in beta-amyloid formation). Liraglutide protects SH−SY5Y neuronal cells from okadaic acid-induced apoptosis, reduces tau activation, and decreases the expression of BACE1 (111). Studies conducted in SH-SY5Y human neuroblastoma cells, a model of neurodegeneration, demonstrated that GLP-1 treatment has multiple effects: both neurotrophic and anti-inflammatory effects and reduction of neurodegeneration. Indeed GLP−1 enhances the survival of SH−SY5Y cells under glutamate- or ROS-induced stress, and it increases the activity of cyclic adenosine monophosphate(cAMP), protein kinase A (PKA), and AMP- activated protein kinase (AMPK). Moreover, it decreases IL−6 levels and TNF−α levels beyond that provides significant protection against toxicity induced by α−synuclein and β−amyloid (112). Using an AD mouse model, Park J.S. et al. highlighted that NLY01, a GLP-1 RA molecule, effectively inhibited microglia (that causes neuronal apoptosis) improving memory and survival of neurons (113). Regarding neuroinflammation, Iwai T. et al., developed a model of neuroinflammation induced by lipopolysaccharide, IL-1β, and hydrogen peroxide (H2O2) and demonstrated that GLP-1 treatment improves synaptic plasticity, preserves cognitive functions and has anti-inflammatory effect (114).

*Exenatide:* Also the exenatide, another GLP1-RA, seems to show effects on neuroinflammation and neuroprotection in animal models of AD. In 5 × FAD transgenic mice treated with exenatide was observed an improvement in cognitive function, a reduction in neuroinflammation and a reduction of oxidative stress. Exenatide exerts anti-inflammatory and neuroprotective effects likely through the inhibition of the NLR family pyrin domain containing 2 (NLRP2) inflammasome in astrocytes. This results in cognitive improvements in animal models of AD and a less pro-inflammatory brain environment (115). Similarly Qian Z. et al. demonstrated that GLP-1R activation reduced microglia-induced neuroinflammation *in vitro* and *in vivo*. Activation of GLP−1R in microglia represents an effective mechanism to reduce neuroinflammation and prevent glial scar formation after central nervous system injuries. The therapeutic effect is partly mediated by the restoration of the PI3K−ARAP3−RhoA pathway, which regulates glial remodelling and inflammatory responses. These findings suggest the potential use of GLP−1R agonists, such as exendin−4, in therapies for spinal cord injuries and possible chronic neuroinflammatory disorders (116). GLP-1 has also effects on neurogenesis. Indeed, in an AD model mice GLP-1 analogues promote neurogenesis in the hippocampus via mitogen activated protein kinases (MAPKs) (117). Zhang L.Q. et al., using a murine hippocampus model with memory deficits caused by neuropathic pain, demonstrated that exenatide acetate (Ex-4) ameliorates memory deficit, reduces inflammatory state by inhibiting the phosphorylation of NF-kB, decreasing the expression of several cytokines (IL-1β and TNF-α), and improving postsynaptic density protein 95 (PSD95), that stimulates synaptic plasticity (118, 119). Two recent reviews have summarized the action of GLP1-RA on neuroinflammation, oxidative stress and neuroprotective functions. Activation of GLP−1R in the brain by GLP-1RA represents an effective intervention against neuroinflammation: it inhibits microgliosis and astrogliosis, suppresses NF−κB and inflammasome activity, reduces peripheral immune cell recruitment, protects against oxidative stress, and promotes neuroprotective signalling (120). Similarly, in a very recent review, De Giorgi R. et al., further confirms that GLP-1 receptor agonists cross the blood–brain barrier and exhibit neuroprotective effects, including: reduction of oxidative stress, improvement of brain insulin signalling, decrease in neuroinflammation, protection against neuronal degeneration. Studies in animal models of Alzheimer’s disease confirm improvements in memory, as well as reductions in amyloid plaques and phosphorylated tau (121). Lastly the neuroprotective effects of GLP-1RA by modulating systemic and brain inflammation were demonstrated also in a recent *post hoc* analysis of the randomized, placebo-controlled EXSCEL trials. In particular treatment with exenatide significantly reduced levels of inflammatory proteins associated with Alzheimer’s disease in patients with type 2 diabetes (122). Moreover a recent systematic review and meta-analysis assessed the impact of different drugs for the treatment of diabetes on the risk of developing dementia, mild cognitive impairment, or cognitive decline highlighting that incretin-based therapies, particularly GLP-1 receptor agonists, were associated with a significant reduction in the risk of cognitive impairment compared to other antidiabetic treatments. This neuroprotective effect is thought to result not only from improved glycemic control but also from direct mechanisms involving modulation of brain inflammation, neuronal protection, and enhanced synaptic plasticity. Metformin, sulfonylureas, insulin, and DPP-4 i studies had inconsistent results. This heterogeneity among included studies emphasized the need for further controlled clinical trials to confirm these findings and explain underlying mechanisms (123).

*Semaglutide:* Very interesting are the preclinical data on cognitive function obtained using the semaglutide, a new GLP-1RA. A recent study investigated the effect of semaglutide on anxious and depressive behaviours as well as cognitive deficits in a mouse model of type 2 diabetes. Administration of semaglutide significantly reduced anxious and depressive behaviours in diabetic mice. It also improved impaired cognitive functions, such as memory and learning. The beneficial effects were mediated through modulation of the microbiota-gut-brain axis. Indeed, semaglutide positively changes the composition of the gut microbiota, reducing systemic and brain inflammation. This contributed to restoring neurochemical balance and brain function (124). The effects of semaglutide on cognitive deficits and glucose metabolism dysfunction in a mouse model of Alzheimer’s diseasewas examined in another preclinical study. Results demonstrated that semaglutide significantly improved the cognitive performance of the mice and corrected alterations in brain glucose metabolism. The beneficial effectswere mediated through the GLP-1R/SIRT1/GLUT4 pathway, which regulates glucose uptake and utilization in the brain, as well as influencing neuroprotective processes (125). A recent study in APP/PS1/tau mice showed that intraperitoneal administration of semaglutide improves working and spatial memory and reduces brain inflammation by promoting the shift of microglia from the pro-inflammatory M1 state to the anti-inflammatory M2 state. Treatment also decreased amyloid-beta deposits and inflammatory cytokines in the hippocampus. These results suggest that semaglutide’s neuroprotective effects involve modulation of microglial activation, adding an important immunoregulatory mechanism to the known metabolic benefits of GLP-1 receptor agonists (126). In contrast, FornyGermano L. et al. evaluated the effects of semaglutide and tirzepatide, a dual agonist GLP-1/GIP receptors, on murine models of Alzheimer’s disease (5XFAD and APP/PS1 mice). However, no significant changes were observed in disease-related pathology, such as amyloid plaques or neurodegeneration. No improvement in behaviour or cognitive functions was found in treated mice compared to controls. It’s true that in these specific Alzheimer’s models, semaglutide and tirzepatide do not appear to affect disease progression or cognitive and behavioural abilities, suggesting that their therapeutic potential may be limited or dependent on the model and experimental conditions (127).

The preclinical studies performed in type 2 diabetes mellitus using the GLP-1 receptor agonist are summarized in the **Table 2**.

**Table 2:** Preclinical studies on cognitive function in T2D using GLP-1 receptor agonist.

Study	Intervention	Outcome	Results
McClean, P.L. et al., (2010) (103)	Administration of GLP-1 analogues	Synaptic plasticity in the brain	GLP-1 analogues enhanced synaptic plasticity, suggesting a potential link between diabetes treatment and neuroprotection in Alzheimer's disease.
Han, W.N. et al., (2013) (104)	Liraglutide treatment in rats exposed to amyloid-β	Spatial learning and memory	Liraglutide protected against amyloid-β-induced impairments in spatial learning and memory in rats.
McClean, P.L. et al., (2011) (105)	Liraglutide treatment in a mouse model of Alzheimer’s disease	Neurodegenerative processes	Liraglutide prevented neurodegeneration and reduced Alzheimer’s-related pathology in mice.
McClean, P.L.; Hölscher, C. (2014) (106)	Liraglutide treatment in aged APP/PS1 mice (Alzheimer’s model)	Memory impairment, synaptic loss, plaque load	Liraglutide reversed memory impairment, reduced synaptic loss, and decreased amyloid plaque load.
Parthsarathy, V.; Hölscher, C. (2013) (107)	Chronic liraglutide administration in an AD mouse model	Cell proliferation and neuronal differentiation	Liraglutide increased neurogenesis by enhancing cell proliferation and differentiation into neurons.
Long-Smith, C.M. et al. (2013) (108)	Liraglutide treatment in a mouse model of Alzheimer’s disease	Insulin receptor signaling, amyloid-β plaque burden, glial pathology	Liraglutide improved insulin receptor localization and signaling, and reduced both amyloid-β plaque load and glial pathology.
Palleria, C. et al. (2017) (109)	Liraglutide treatment in a rat model of streptozotocin-induced diabetes	Cognitive function	Liraglutide prevented cognitive decline independently of its peripheral metabolic effects.
Candeias, E. et al. (2018) (110)	Exendin-4 treatment in type 2 diabetic rats	Neuronal apoptosis, GLP-1/IGF-1 signaling, autophagy	Exendin-4 protected against neuronal apoptosis via brain GLP-1/IGF-1 signaling and autophagy mechanisms.
Yu, C.J. et al. (2020) (111)	Use of GLP-1/GIP receptor agonists in Alzheimer’s disease models	Alzheimer’s pathology and neurodegeneration	GLP-1/GIP receptor agonists showed potential to improve cognitive function and reduce AD-related pathology.
Li, Y. et al. (2021) (112)	GLP-1 (9-36) metabolite in cellular models of neurodegeneration	Neuroinflammation and neuroprotection	GLP-1 (9-36) demonstrated neuroprotective and anti-inflammatory effects in neurodegeneration models.
Park, J.S. et al. (2021) (113)	Blocking microglial activation of reactive astrocytes in Alzheimer's models	Neuroprotection	Inhibiting microglial activation of astrocytes was neuroprotective, reducing neurodegeneration in Alzheimer’s models.
Iwai, T. et al. (2014) (114)	GLP-1 administration in rodents with neuroinflammation	Synaptic and learning functions	GLP-1 preserved synaptic integrity and learning ability against neuroinflammatory damage.
Zhang, M. et al. (2022) (115)	GLP-1 analogs in Alzheimer’s disease models	Astrocyte-mediated neuroinflammation	GLP-1 analogs suppressed NLRP2 activation in astrocytes, mitigating neuroinflammation.
Qian, Z. et al. (2022) (116)	GLP-1 receptor activation in microglia after nerve injury	Glial scarring, neuroinflammation	GLP-1R activation reduced glial scarring and neuroinflammation by restoring ARHGAP3 expression.
Hamilton, A.; Holscher, C. (2012) (117)	Aging effects studied in APP/PS1 Alzheimer’s mouse model	Neurogenesis and oxidative stress	Aging was associated with reduced neurogenesis and increased oxidative stress in the Alzheimer’s model.
Zhang, L.Q. et al. (2021) (118)	GLP-1R activation in neuropathic pain mice	Novel-object recognition memory, AMPK/NF-κB pathway	GLP-1R activation improved memory by modulating the hippocampal AMPK/NF-κB signaling pathway.
Meyer, D. et al. (2014) (119)	Study of synaptic plasticity mechanisms	Synaptic structure balance and stability	Demonstrated that synaptic structures maintain balance and stability during plasticity, though not directly focused on GLP-1.
Diz-Chaves, Y. et al. (2022) (120)	GLP-1R activation in neurodegenerative disease models	Brain inflammation	GLP-1R activation exerted strong anti-inflammatory effects in the brain in various neurodegenerative conditions.
De Giorgi, R. et al. (2025) (121)	Review of GLP-1 receptor agonists in major neurocognitive disorders	Cognitive decline, dementia	Evidence suggests GLP-1R agonists may have beneficial effects on cognition in neurocognitive disorders, including Alzheimer’s.
de Paiva, I.H.R. et al. (2024) (122)	Semaglutide treatment in T2DM mouse model	Anxiety, depression, cognitive impairment	Semaglutide improved mood-related behaviors and reversed cognitive impairment via the microbiota-gut-brain axis.
Wang, Z.J. et al. (2023) (125)	Semaglutide in 3xTg mouse model of Alzheimer’s disease	Cognition, glucose metabolism	Semaglutide improved cognition and glucose metabolism through the GLP- Preclinical 1R/SIRT1/GLUT4 Preclinical pathway.
Forny Germano, L. et al. (2024) (127)	Semaglutide and tirzepatide in 5XFAD and APP/PS1 mice	AD pathology, behavior, cognition	Neither semaglutide nor tirzepatide altered disease pathology, behavior, or cognitive function in these Alzheimer’s models.

### 5.2 Clinical studies

Regarding clinical evidence of GLP-1 effects on cognitive function, there are less evidence than *in vivo* and *in vitro* studies but several trials in humans are ongoing in humans and there is currently an increasing interest on these drugs although the study results are conflicting.

*Exenatide:* In 2019, the results of a pilot study on exenatide action in AD were published. The study demonstrated that exenatide was safe in patients with early Alzheimer’s disease and seems to have a biological effect (reduction of Aβ in extracellular vesicles), but it does not show cognitive or clinical benefits. However, the study was underpowered due to early termination and therefore the authors could not draw any firm conclusions (128). Similarly, in a study where 20 obese patients with schizophrenia were treated with exenatide (once weekly for 3 months), GLP-1 RA had no significant effects on cognitive functions (129).

*Liraglutide:* Conflicting results have also been obtained with liraglutide. A 26-week double-blinded, randomized clinical trial including 38 subjects with early/moderate AD evaluated the effects of liraglutide compared to placebo on cognitive function. Researchers used Pittsburgh compound B (PIB) positron emission tomography (PET) scans to evaluate the changes in the intracerebral amyloid deposits in the central nervous system. No changes in amyloid deposition or cognition were highlighted, but it may have been due to a short follow-up time (130). Another phase 2 multicenter, randomized, double-blinded study (ELAD study) involved always liraglutide. The ELAD study has enrolled 204 subjects with mild AD treated exactly with liraglutide or matching placebo. It was a robust protocol for a 12-month clinical trial designed to assess whether liraglutide was able to improve metabolic and cognitive decline in mild Alzheimer’s disease, using a range of endpoints including neuroimaging, biomarkers, neuroinflammation, and cognitive function. Indeed, the primary outcome was the change in cerebral glucose metabolic rate from baseline to follow-up in two groups. The secondary outcomes were change in clinical and cognitive scores; change in magnetic resonance imaging volume; reduction in microglial activation and in tau formation; change in amyloid levels. The ELAD study is closed, and results highlighted that liraglutide induces changes in the brain but does not improve cognitive measures (131). More encouraging results were obtained in subjects with prediabetic conditions or type 2 diabetes mellitus. In a study by Valdini F. et al. forty (40) subjects with prediabetic conditions or with new onset T2DM received liraglutide (1.8 mg daily) or lifestyle counselling. The aim was to evaluated whether treatment with GLP-1RA could improve cognitive functions in prediabetic or diabetic subjects. The results showed that liraglutide slows down cognitive decline as reported by a psychological test evaluating attention, memory, and executive control (132). Similarly, in a prospective, parallel-group, open- label, phase III study was evaluated whether liraglutide could improve cognitive function in diabetic patients and if such improvement was associated with metabolic changes. Patients,with inadequately control with oral antidiabetic drugs or insulin, were randomized to receive liraglutide or placebo + standard of care group for 12 weeks. Patients assessments were performed by functional near-infrared spectroscopy (fNIRS) to monitor brain activation and neuropsychological cognition tests to evaluate memory, executive function, attention and verbal function. At the end of study, the liraglutide group achieved better scores in all cognitive tests, notably in memory and attention compared to control group. Moreover, liraglutide increased activation of the dorsolateral prefrontal cortex and orbitofrontal cortex. All the cognitive improvement in this group was correlated with morphological changes in brain regions. All these results are metabolism-independent, meaning they are not related to hypoglycemia or weight loss. The results suggest that liraglutide could be a targeted intervention for early cognitive decline, independent of its effects on metabolism (133). In 2018 Wu et al., enrolling 106 patients with T2DM and evaluating the association between serum GLP-1 concentration and mild cognitive function impairment in MCI, demonstrated that lower serum GLP-1 levels are correlated to cognitive dysfunction in patients with diabetes (134). In another randomized, prospective, open-label, parallel-group trial the comparative effects of liraglutide, dapagliflozin and acarbose in patients with T2DMhas been evaluated. The subjects inadequately controlled with metformin, were randomized to receive for 16 weeks liraglutide, dapagliflozin or acarbose. At baseline and post treatment, all participants performed a brain functional magnetic resonance imaging (MRI) scan and subjected to cognitive function assessments tests(such as Mini-Mental State Examination, MMSE, Montreal Cognitive Assessment, MoCA). The results showed that in the group treated with liraglutide, a statistically significant improvement in impaired odor-induced left hippocampal activation and an improvement of cognitive subdomains of delayed memory, attention and executive function was observed (135).

*Dulaglutide*: Recently, a *post-hoc* analysis of a phase 3 randomized, double-blinded, placebo-controlled trial (REWIND) assessed the effect of dulaglutide on cognitive impairment (CI) in T2DM. This study involved 9901 participants, 4949 randomized to receive dulaglutide and 4952 placebo. Follow-up lasts 5.4 years and cognitive function was evaluated only in 8828 subjects (4456 received dulaglutide and 4372 received placebo) using the MoCA and Digit Symbol Substitution Test (DSST). CI occurred in 4·05 per 100 patient-years in dulaglutide group and 4·35 per 100 patient-years in placebo one, but after *post-hoc* adjustment, the risk of CI in people with T2DM aged 50 years or above who had further cardiovascular risk factors was reduced by 14% of subjects in the dulaglutide arm (HR 0.86, 95% confidence interval 0.79–0.95; *p* = 0.0018). This analysis showed that long-term treatment with dulaglutidemay reduce risk of CI in patients with T2DM (136).

*Semaglutide*: Similarly, also the effects of semaglutide on cognitive functions are ongoing.Evoke and Evoke+ are two large scale, double- blind, placebo-controlled, phase III trials evaluating efficacy, safety, and tolerability of oral semaglutide in early-stage symptomatic AD. The primary endopoint is to evaluate whether semaglutide modifies biomarkers and improves neuroinflammation. The trials are ongoing and completion is expected in September 2025 (137). Interestingly, Wang W. et al., conducted a target trial emulation using nationwide real-world data of 116 million US patients, evaluating the association of semaglutide with AD diagnosis in patients with T2DM. The study explored whether semaglutide is linked to a lower risk of developing Alzheimer’s disease compared to other diabetes treatments. The results suggest that semaglutide may reduce the risk of a first-time Alzheimer’s diagnosis, indicating its potential neuroprotective effects beyond blood glucose management, which could help delay or reduce the onset of neurodegenerative diseases like Alzheimer’s (138). Current innovations—such as dual and triple agonists, oral formulations, and less frequent dosing—mark a cultural shift, aiming to expand access and enhance effectiveness in tackling obesity, chronic diseases, and metabolic disorders. In particular they underline neuroprotective effects: observational studies suggest a reduction in the risk of dementia by up to 25% compared to metformin in elderly patients with type 2 diabetes, possibly due to the ability to cross the blood-brain barrier and modulate brain inflammation (139). Another 2024 review underlines Neuroprotection effect of GLP-1 RA. Observational studies link the use of GLP−1 receptor agonists to a reduced risk of Alzheimer’s disease, dementia, ischemic stroke, and mortality—by as much as 45% in some studies (140). The clinical studies performed in type 2 diabetes mellitus using the GLP-1 receptor agonist are summarized in the **Table 3**.

**Table 3:** Clinical studies on cognitive function in T2D using GLP-1 receptor agonist.

Study	Intervention	Outcome	Results
Mullins, R.J. et al. (2019) (128)	Exenatide treatment in Alzheimer’s disease patients (pilot study)	Cognitive function and biomarker levels	Exenatide was well tolerated and showed trends toward improved biomarker profiles, warranting further investigation.
Ishøy, P.L. et al. (2017) (129)	GLP-1 receptor agonist (liraglutide) in antipsychotic-treated, obese patients with schizophrenia	Cognitive performance	No cognitive-enhancing effect was observed with GLP-1 receptor agonism in this patient population.
Gejl, M. et al. (2016) (130)	6-month treatment with GLP-1 analogue in AD patients (RCT)	Brain glucose metabolism	GLP-1 analogue prevented the decline in brain glucose metabolism in patients with early-stage Alzheimer’s disease.
Femminella, G.D. et al. (2019) (131)	Liraglutide in Alzheimer’s disease (ELAD study protocol)	Cognitive and functional outcomes	Protocol paper—describes a randomized trial designed to assess liraglutide’s effects on cognition and function in AD (results not reported).
Vadini, F. et al. (2020) (132)	Liraglutide treatment in obese patients with prediabetes or early T2DM	Memory performance	Liraglutide significantly improved memory performance in these patients.
Li, Q. et al. (2021) (133)	GLP-1R activation in T2DM patients	Cognitive decline	GLP-1R activation ameliorated cognitive decline through a pathway independent of glucose metabolism.
Wu, P. et al. (2018) (134)	Measurement of serum GLP-1 levels in T2DM patients	Mild cognitive impairment (MCI)	Low serum GLP-1 levels were associated with the presence of MCI in patients with type 2 diabetes.
Cheng, H. et al. (2022) (135)	Liraglutide vs. dapagliflozin vs. acarbose in T2DM patients (16-week RCT)	Cognitive capacity and olfactory activation	Liraglutide improved both olfactory neural activation and cognitive performance, unlike the other two drugs.
Cukierman-Yaffe, T. et al. (2020) (136)	Dulaglutide (GLP-1 RA) in the REWIND trial (exploratory cognitive analysis)	Cognitive impairment in T2DM patients	Dulaglutide modestly reduced cognitive decline in older adults with type 2 diabetes.
Cummings, J.L. et al. (2025) (137)	Semaglutide in early-stage Alzheimer’s disease (EVOKE and EVOKE+ Phase 3 trials)	Cognitive function, safety, and tolerability	Study design paper—aims to evaluate the efficacy and safety of semaglutide in early symptomatic AD. Results pending.
Wang, W. et al. (2024) (138)	Semaglutide use in T2DM patients (real-world data study)	First-time Alzheimer’s diagnosis	Semaglutide use was associated with a reduced risk of first-time Alzheimer's diagnosis in patients with T2DM.
Zheng, Z. et al. (2024) (139)	Review of GLP-1 receptor mechanisms and therapeutic applications	GLP-1 receptor-related effects on neurological and metabolic health	Highlights GLP-1R's role in neuroprotection, anti-inflammation, and cognitive preservation mechanisms.
Olukorode, J.O. et al. (2024) (140)	Review of GLP-1 agonists in T2DM and metabolic disorders	Cognitive and metabolic outcomes	GLP-1 agonists offer therapeutic benefits beyond glycemic control, including potential improvements in cognitive and cardiovascular health.

## 6 Neuroprotective effect of GLP-1/GIP dual receptor agonist

Tirzepatide (TRZ) acts as a dual agonist on GLP-1 and GIP receptors and can affect several molecular pathways in nervous system, such as directly Aβ-induced neurodegeneration or indirectly by reducing brain insulin resistance (BIR) (141). Furthermore, TRZ affects oxidative stress, neuroinflammation and brain leptin resistance in obesity (142). Literature confirmed that tirzepatidecan considerably activate the CREB/BDNF signalling cascade. CREB, that is a member of transcription factors, acts on nervous system growth and development, synaptic plasticity and long-term memory formation (145–148). Moreover, it controls neuronal survival through the transcription of BDNF, that is a neurotrophin that acts on the survival and differentiation of neurons (149). Further actions are anti-apoptotic process (BAX/Bcl-2), neurodifferentiation (phosphorylated protein kinase: pAkt, microtubule-associated protein-2: MAP2, growth-associated protein-43: GAP43 and ATP/GTP binding protein-like 4: AGBL4) and neuronal glucose homeostasis (Glucose transporter 1, 3, 4: GLUT1, GLUT3 and GLUT4). Furthermore, TRZ affects BIR by regulating the expression of PI3K/AKT/GSK3β, that is a main regulator of brain insulin signalling and controls the functional activity of astrocytes and microglia (150). Moreover, by modulating CREB, tirzepatide is able to regulate the expression of the anti-apoptotic gene B Cell Lymphoma-2 (Bcl-2) expression (151), reducing caspase-3 and BAX activity while simultaneously increasing Bcl-2 activity (143, 144). In fact an augment in BAX/Bcl-2 ratio results in memory loss and learning capacity deregulation (152). TRZ modulates the pAkt/CREB/BDNF, modifying, in turn, neuronal differentiation markers like MAP2, GAP- 43 and AGBL4 (153–156). TRZ, activating GLP1 receptor, avoided the down-regulation of GLUT3, GLUT1, GLUT4, and Sorbin and SH3 domain-containing protein 1 (SORBS1) amending neuronal insulin resistance (157). Finally, it can regulate epigenetic modulators of neuronal growth (miRNA 34a), apoptosis (miRNA 212), and differentiation (miRNA 29c) (158). In a very recent preclinical study Ma et al., investigated the effects of a novel dual GLP 1/CCK receptor agonist in the 5×FAD mouse model of Alzheimer’s disease. This drug, compared with Liraglutide (50 nmol/kg ip.) as a positive control, significantly improved cognitive performance (both spatial and working memory), reduced beta-amyloid deposition, and attenuated neuroinflammatory markers such as NLRP3 and TNF α. On a molecular level, treatment enhanced BDNF and TrkB expression, promoted synaptogenesis, and activated the PI3K AKT pathway—known for its neurotrophic and neuroprotective properties. However, not all improvements were superior to Liraglutide, suggesting that the added benefit from CCK activation needs further validation. Notably, in other outcomes, the dual agonist outperformed liraglutide, suggesting that simultaneous activation of multiple gut hormone receptors may offer greater neuroprotective potential in neurodegenerative diseases (159). The preclinical studies performed in type 2 diabetes mellitus using the dual agonist GLP-1R/GIPR are summarized in the **Table 4**.

**Table 4:** Preclinical studies on cognitive function in T2D using the dual agonist GLP-1R/GIPR (Tirzepatide).

Study	Type of study	Intervention	Outcome	Results
Alshehri G.H. et al. (2025) (141)	Preclinical	Tirzepatide as a potential therapy for Alzheimer’s disease	Therapeutic potential and mechanisms in AD	Tirzepatide shows promise due to its anti-inflammatory and neuroprotective effects, suggesting it may be a novel therapeutic option for Alzheimer’s disease.
Ma, J. et al. (2025) (142)	Preclinical	Tirzepatide in high-fat diet (HFD)-induced cognitive impairment in mice	Cognitive function, neuroinflammation	Tirzepatide improved cognition by regulating the SIRT3–NLRP3 axis, reducing neuroinflammation and oxidative stress.
Fontanella RA et al. (2024) (143)	Preclinical	Tirzepatide administration	Neurodegeneration prevention	Tirzepatide prevents neurodegeneration by modulating multiple molecular pathways, including inflammation and apoptosis.
Chen J et al. (2018) (144)	Preclinical	GLP-1 receptor regulation in chondrocytes under ER stress	Apoptosis and inflammation in osteoarthritis	GLP-1 receptor activation reduced ER stress-induced apoptosis and inflammation, slowing osteoarthritis progression.

## 7 Conclusions and future perspective

Recent evidence on the use of incretins offers new perspectives in the treatment and notably in the prevention of cognitive decline. Although some studies are still in the preliminary stages, the results seem to be comparable and suggest that these drugs, originally developed for T2DM, may exert significant neuroprotective effects, improving cognitive function and slowing the progression of neurodegenerative diseases such as Alzheimer’s. These effects result from mechanisms such as the reduction of brain inflammation, the improvement of synaptic plasticity, and neuronal protection. However, further clinical and preclinical studies are essential to confirm these results and fully understand the therapeutic potential of incretins in the context of cognitive decline. The integration of innovative pharmacological approaches could help in the fight against neurodegenerative diseases, not only for diabetic patients but also for the general population.

## Glossary

AD: Alzheimer’s disease
AGBL4: ATP/GTP binding protein-like 4
AGEs: advanced glycation end products
AMPK: AMP-activated protein kinase
APOE ϵ4: Apolipoprotein ϵ4
APP/PS1: amyloid precursor protein/presenilin-1
APP: amyloid precursor protein
Aβ: β-amyloid
BACE-1: β-site amyloid precursor protein cleaving enzyme-1
BBB: blood–brain barrier
Bcl2: B-cell lymphoma 2
BDNF: brain-derived neurotrophic factor
BIR: brain insulin resistance
CDK5: cyclin-dependent kinase 5
CI: cognitive impairment
cAMP: cyclic adenosine monophosphate
CREB: cAMP-response element binding protein
DCCT: Diabetes Control and Complications Trial
DPP-4: dipeptidyl peptidase IV
DPP4-i: dipeptidyl peptidase-4 inhibitors
DSST: Digit Symbol Substitution Test
Ex-4: Exendin-4
fNIRS: functional near-infrared spectroscopy
GAP-43: growth-associated protein-43
GIP: gastric inhibitory polypeptide
GIPR: GIP receptor
GLP-1: glucagon-like peptide-1
GLP-1R: glucagon-like peptide-1 receptor
GLP-1RA: GLP-1 receptor agonist
GLUT1: glucose transporter 1
GLUT3: glucose transporter 3
GLUT4: glucose transporter 4
GSK-3β: glycogen synthase kinase 3 beta
H2O2: hydrogen peroxide
HbA1c: glycated haemoglobin
HRs: hazard ratios
ICAM: intercellular adhesion molecule 1
IDE: insulin-degrading enzyme
IGF1: insulin-like growth factor 1
IGF2: insulin-like growth factor 2
IL-1β: interleukin-1β
IRS-1: insulin receptor substrate-1
JNK: c-Jun N-terminal kinase
L-LTP: late-phase long-term potentiation
LTP: long-term potentiation
MAP2: microtubule-associated protein-2
MAPKs: mitogen-activated protein kinases
MCI: mild cognitive impairment
MCP-1: macrophage chemoattractant protein 1
MIP-1α: macrophage inflammatory protein-1α
MMSE: Mini-Mental State Examination
MoCA: Montreal Cognitive Assessment
MRI: magnetic resonance imaging
NC: normal cognitive function
NFTs: neurofibrillary tangles
NLRP2: NLR family pyrin domain containing 2
Nrf2: nuclear factor erythroid 2-related factor 2
pAkt: phosphorylated protein kinase B
PET: positron emission tomography
PGC-1α: peroxisome proliferator-activated receptor gamma coactivator 1-alpha
PI3K/Akt: phosphoinositide 3-kinase/protein kinase B
PIB: Pittsburgh compound B
PKA: protein kinase A
PSD95: postsynaptic density protein 95
RAGEs: receptors for advanced glycation end products
ROS: reactive oxygen species
SH-SY5Y: human neuroblastoma cell line
SIRT1: sirtuin 1
SOD: superoxide dismutase
SORBS1: Sorbin and SH3 domain-containing protein 1
STZ: streptozotocin
T1DM: Type 1 diabetes mellitus
T2DM: Type 2 diabetes mellitus
TNF-α: tumor necrosis factor-α
TRZ: Tirzepatide
VaD: vascular dementia
VCAM-1: vascular cell adhesion molecule 1
